# Chikungunya Fever in Travelers Returning to Europe from the Indian Ocean Region, 2006

**DOI:** 10.3201/eid1403.070906

**Published:** 2008-03

**Authors:** Marcus Panning, Klaus Grywna, Marjan van Esbroeck, Petra Emmerich, Christian Drosten

**Affiliations:** * Bernhard Nocht Institute for Tropical Medicine, Hamburg, Germany; † Prince Leopold Institute of Tropical Medicine, Antwerp, Belgium; 1 Current affiliation: University of Bonn Medical Centre, Bonn, Germany.

**Keywords:** chikungunya, travelers, dengue, arbovirus diagnostics, real-time RT-PCR, serology, research

## Abstract

Chikungunya fever has spread through several Indian Ocean islands and India, including popular travel destinations. To compare usefulness of diagnostic tests and to understand reasons for the magnitude and severity of an outbreak, we used 3 diagnostic methods to test 720 samples from 680 patients returning to Europe from the Indian Ocean region in 2006. Chikungunya infection was confirmed for 24.4% patients in the first half of the year and for 9.9% in the second half. Reverse transcription–PCR was positive for all samples taken up to day 4 after symptom onset. Immunofluorescence detected immunoglobulin (Ig) M on day 1 and IgG on day 2 for some patients, and in all patients from day 5 onward. Soon after onset of symptoms, patients had IgG and IgM and high viral loads (some >10^9^ copies/mL plasma). These data will help healthcare providers select diagnostic tests for returning travelers.

## Abstract 2

Chikungunya fever should be added to the list of differential diagnoses for ill travelers returning from this region.

In 2005 and 2006 an outbreak of chikungunya fever of unprecedented magnitude spread over the western Indian Ocean region, including the Comoros Islands, Mauritius, Réunion Island, Madagascar, and the Seychelles (*1*,*2*). By October 2006 on Réunion Island alone, which has a population of 760,000, at least 266,000 cases had been reported (*3*). The epidemic swept eastward into the Indian subcontinent, where by the end of the year it had caused >1.3 million cases; attack rates were 45% in some regions (*4*,*5*). By the beginning of 2007, the epidemic was on the decline on La Réunion and the Seychelles (*6*), but it seems to be continuing in areas of India. New outbreaks have been reported from early 2007 in Malaysia and mid 2007 in Indonesia (*7*,*8*).

*Chikungunya virus* (CHIKV) is an arthropod-borne RNA virus of the genus *Alphavirus,* family *Togaviridae*. Its genome is single stranded, of positive polarity, and 11.7 kb long. Based on partial sequences of the envelope protein E1, CHIKV strains can be grouped into 3 distinct genetic lineages, which share a common ancestor in tropical Africa (*9*). The virus is transmitted to humans by numerous *Aedes* mosquito species, including *Ae. aegypti* and *Ae. albopictus* (*2*,*10*–*12*). The latter is thought to be less competent as a vector (*2*).

Strict mosquito-control measures in 2006 ameliorated the outbreaks on the Indian Ocean islands, but the spread of the same strain of CHIKV to India proves that the virus is not easy to contain (*2*,*5*). Tourists visiting these regions have imported the virus back to Europe and the United States, including regions of these countries in which the vector is known to be present (*13*,*14*).

CHIKV infection in humans is characterized by a sudden onset of fever, rash, and severe arthralgia (*15*–*17*). No specific treatment exists and symptoms are generally self-limiting (*16*,*18*). Because in popular tourist destinations in the Indian Ocean region, the disease is endemic, along with malaria and dengue, CHIKV testing is now being conducted in outpatient settings. Antibody assays, virus isolation, and reverse transcription–PCR (RT-PCR) are available (*19*–*21*).

For clinical management of chikungunya, knowing which laboratory assays provide what information at given points of time during disease is helpful; cross-sectional detection rates and kinetics of virologic parameters over time (virus RNA, immunoglobulin [Ig] M, and IgG) are crucial. Unfortunately, these data for CHIKV are minimal because studies on large patient cohorts were completed before relevant laboratory tests, particularly RT-PCR, became available. More recent studies have used such methods, but the numbers of studied patients have been limited (*14*,*22*,*23*). To provide support for the selection of diagnostic tests, we collected a cumulative figure of virologic parameters (viral RNA detection and antibody testing results over time) for the largest cohort of returning travelers studied to date. In addition, we sought possible reasons for the magnitude and severity of current outbreaks. Of relevance is a recent finding that Indian Ocean CHIKV strains display genetic characteristics in their structural E1 gene (*22*), especially at amino acid position 226. Similar to the related Semliki Forest virus, in which a homologous mutation causes enhanced membrane fusion capacity in insect cells, the virus could have an advantage in insects or even in humans (*22*,*24*). We determined whether the mutation had an influence on viral loads in the patients in our cohort and how it was distributed geographically and temporally during 2006.

## Materials and Methods

### Patients and Clinical Samples

From January 1 through December 31, 2006, we tested 720 samples from 680 patients at the Bernhard-Nocht Institute for Tropical Medicine in Hamburg, Germany, for CHIKV infection. All had symptoms compatible with acute or recent CHIKV infection (sudden onset of fever, muscle and joint pain, headaches, rash) upon return to Europe (Germany, n = 515; Belgium, n = 99; Switzerland, n = 42; Denmark, n = 22; Poland, n = 2). For 189 patients, exact travel destinations were reported: Madagascar (n = 9), Mauritius (n = 92), the Seychelles (n = 23), Réunion Island (n = 18), Bali (n = 2), Indonesia (n = 6), Sri Lanka (n = 5), India (n = 28), Malaysia (n = 2), Kenya (n = 1), and Thailand (n = 3). Average ages of travelers to each country did not differ significantly (analysis of variance F-test, p>0.05). For 121 patients, exact dates of onset and sampling could be retrieved through voluntary questionnaires completed by telephone or fax after issuance of results. Age and sex distribution and travel histories for these patients are shown in Table 1. The day of onset of symptoms was defined as day 0. All samples with possible CHIKV in 2006 were tested for IgG and IgM by indirect immunofluorescence. During the first half of 2006, all samples were tested by real-time RT-PCR in addition; however, in the second half of the year RT-PCR testing was restricted to samples from patients with acute infection only (on the basis of experiences from the first 6 months as described below). Classification of patients as having laboratory-confirmed cases required either a positive RT-PCR or IgM result or an isolated IgG detection with at least a subsequent 4-fold increase in titer. The Statgraphics Plus 5.1 software package (Manugistics, Dresden, Germany) was used for all statistical analyses.

**Table 1:** Characteristics of chikungunya fever patients  for whom travel and disease histories were known

Location visited	Age, y			Sex	
	Mean	Range		M	F
Mauritius (n = 69)	48.2	16–72		28	41
Seychelles (n = 17)	47.9	24–74		7	10
India (n = 15)	45.6	22–76		5	10
Réunion Island (n = 10)	47.3	17–78		3	7
Madagascar (n = 5)	45.2	29–53		4	1
Sri Lanka (n = 3)	38.7	34–48		3	0
Kenya (n = 1)	47	NA*		1	0
Indonesia (n = 1)	56	NA		0	1
Total (n = 121)	47.4	16–78		51	70

*NA, not applicable.

### Indirect Immunofluorescence

CHIKV strain S27 was grown on Vero cells at a multiplicity of infection of 0.5. Cells were spread on slides after 24 h, air dried, and fixed in ice-cold acetone. Serum samples were incubated for 1 h (IgG) or overnight (IgM) on fixed cells. Antibodies were detected by anti-human IgG or IgM labeled with fluorescein isothiocyanate (Sifin, Berlin, Germany). For IgM testing, IgG was absorbed before testing by Biosorb resin (Biomed, Munich, Germany). Specificity of antibodies was confirmed by plaque-reduction neutralization assay on a selection of samples (data not shown).

### Nucleic Acid Testing

Two versions of real-time RT-PCR targeting the *nsp1*gene of CHIKV were used: 1 for CHIKV in general and 1 adapted specifically to the current epidemic strain. Reaction conditions and oligonucleotides are listed in Table 2. Quantified in vitro–transcribed RNA was generated by cloning the RT-PCR target region in plasmid pCR2.1 (Invitrogen, Karlsruhe, Germany). Inserts together with a 5′-T7 promotor sequence were amplified with M13 plasmid-specific primers and transcribed into RNA by use of Megascript T7 reagents (Ambion, Austin, TX, USA). After DNase digestion and affinity purification, transcripts were quantified by photometer and used as quantification standards.

**Table 2:** Real-time reverse transcription–PCR (RT-PCR) assay results for chikungunya virus (CHIKV)

Oligonucleotide name	Purpose*	Sequence and label, 5′ →3′	Position (GenBank accession no.)
ChikSI	Forward primer, general CHIKV assay	TGATCCCGACTCAACCATCCT	241-261 (AF369024)
ChikSII	Forward primer, adapted assay for Indian Ocean strain	CCGACTCAACCATCCTGGAT	246-265 (DQ443544)
ChikAsI	Reverse primer, general CHIKV assay	GGCAAACGCAGTGGTACTTCCT	323-302 (AF369024)
ChikAsII	Reverse primer, adapted assay for Indian Ocean strain	GGCAGACGCAGTGGTACTTCCT	323-302 (DQ443544)
ChikP	Detection probe, CHIKV	FAM-TCCGACATCATCCTCCTTGCTGGC-BHQ1	300-277 (AF369024)
ICP	Detection probe, internal control	DYXL-ATCGTTCGTTGAGCGATTAGCAG-BHQ2	Not applicable

*All oligonucleotides were used in the following assay: 25-µL reaction volume, 3 µL of RNA extract (Viral RNA Mini Kit, QIAGEN, Hilden, Germany), QIAGEN OneStep RT-PCR Kit, 600 nmol/L each primer, 200 nmol/L each probe. Cycling at 50°C for 30 min, 95°C for 15 min, 45 cycles each at 95°C for 15 s and 58°C for 30 s, LightCycler (Roche, Mannheim, Germany).

Competitive internal controls were generated by overlap-extension PCR as described (*25*,*26*), by using ChikS/ChikAs primers and mutagenic primers 5′- ATCGTTCGTTGAGCGATTAGCAGCAGGAAGTACCACTGCGTTTGCC-3′and 5′- CTGCTAATCGCTCAACGAACGATGCACTACCAATATCCAGGATGGTTG-3′. Mutated constructs were cloned into pCR2.1 and transcribed into RNA. Both assays did not cross-react with RNA extracted from serum samples with high titers or undiluted cell culture supernatants of the following viruses: Barmah Forest, dengue, Epstein-Barr, hepatitis C, herpes simplex type 1, human immunodeficiency type 1, Japanese encephalitis, poliomyelitis type 1, Ross River, Sindbis, Venezuelan equine encephaltitis, West Nile, and yellow fever. According to series of parallel limiting-dilution experiments and probit analysis (*25*,*26*), the general CHIKV assay detected 3,844 RNA copies/mL of serum at >95% certainty (95% confidence interval [CI] 2,834–6,832 copies/mL); the assay adapted to the current Indian Ocean strain detected 2,285 copies/mL at 95% certainty (95% CI 1,694–5,326 copies/mL) (online Appendix, available from www.cdc.gov/EID/content/14/3/416-appG1.htm). On the whole panel of clinical samples described in this study, quantitative correlation between both assays was close (regression analysis slope = 1, SD 0.04, p<0.001; online Appendix, available from www.cdc.gov/EID/content/14/3/416-appG2.htm).

### Sequence Analysis

A fragment of the CHIKV E1 gene (positions 10265–11158, GenBank accession no. DQ443544) was amplified directly from patient plasma and sequenced. Sequencing was performed with a CEQ 8000 Genetic Analysis System (Beckman Coulter, Krefield, Germany) as recommended. Sequences were analyzed with Lasergene software package (DNASTAR, Madison, WI, USA).

### Dengue Virus Testing

Serum samples were tested for dengue antibodies by an IgM µ-capture assay (Pan-Bio, Sinnamon Park, Queensland, Australia) and by indirect IgG immunofluorescence assay as described (*27*). All samples that were reportedly taken during the first 12 days of illness were also tested for dengue virus by real-time RT-PCR (*28*).

## Results

### Cross-sectional Laboratory Results

CHIKV infection was laboratory-confirmed for 152 (22%) symptomatic patients and 188 (26%) samples. Median IgG titer for all 156 IgG-positive samples was 2,560 (range 40–40,960). Median IgM titer for 136 IgM-positive samples was 320 (range 20–10,240). Median viral load for 50 RNA positive samples was 1.7 × 10^5^ RNA copies/mL (range 1 × 10^3^–1.2 × 10^10^ RNA copies/mL). Table 3 summarizes cross-sectional testing results for individual patients at time of first visit.

**Table 3:** Detection of chikungunya virus infections in patients at initial examination, by laboratory test, 2006*

Period	IgG and IgM			IgG only			IgM only			RT-PCR†			RT-PCR only	
	No. (%)	Total		No. (%)	Total		No. (%)	Total		No. (%)	Total		No. (%)	Total
Jan–Jun	85 (23.1)	368		5 (1.4)	368		2 (0.5)	368		42 (11.8)	355		9 (2.5)	355
Jul–Dec	13 (4.2)	312		9 (2.9)	312		0	312		6 (30)	20		0	20
Total	98 (14.4)	680		14 (2.1)	680		2 (0.3)	680		48 (12.8)	375		9 (2.4)	375

*Ig, immunoglobulin; RT-PCR, reverse transcription–PCR. †Some patients were tested by using both RT-PCR and immunofluorescence; only RT-PCR results are given.

In the first half of the year, the rate of confirmed infection for patients with suspected CHIKV was 24.4%; in the second half, 9.9%. Although the numbers of laboratory tests were comparable in the first and second half of the year (396 vs. 326), laboratory confirmation rates in travelers were significantly higher in the first half (χ^2^ test, p<0.001).

### Cumulative Antibody Kinetics

**Figure 1:**
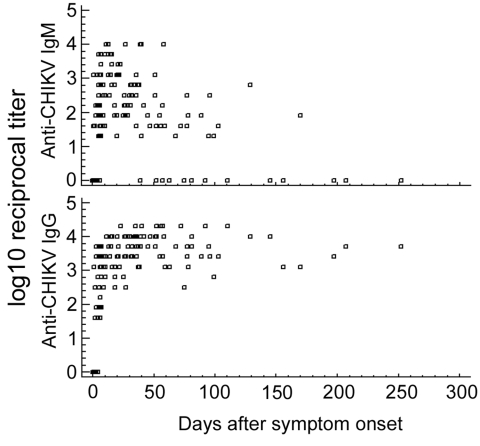
Immunoglobulin (Ig) M and IgG titers in 153 samples from 121 patients. Some patients are represented more than once if multiple specimens were submitted for testing. CHIKV, chikungunya virus.

We were able to retrieve 153 samples, exact information on travel destination, onset of symptoms, and date of sampling for 121 patients (Figure 1). CHIKV IgG was found for 79% of patients (125 [81.7%] of 153 samples). The earliest sample with a detectable IgG titer had been drawn on day 2 after onset; the latest, on day 252. An isolated IgG titer, i.e., no concomitant IgM, was detected in 15 samples, 14 of which had been collected late after onset (median 86.5 days, range 39–252 days). The remaining sample, collected on day 2, was positive by RT-PCR.

Demonstrable CHIKV IgM was found in 111 (72.5%) of 153 samples from 88 (72.7%) patients. The earliest IgM-positive result was obtained from a sample drawn on day 1; the latest was obtained on day 170, from 1 sample. The only IgM sample with no concomitant IgG titer was drawn on day 1 after onset of fever; it was concomitantly positive by RT-PCR.

For 11 patients whose first sample yielded antibodies, consecutive samples were available. All paired samples were drawn at least 14 days apart. The first samples were collected after a median of 13 days from onset (range 5–68 days), and all had IgM (median titer 320) and IgG (median titer 2,560). The second samples were collected after a median of 77 days from onset (range 20–207 days). Median IgG titer was 5,120; median IgM titer was 40. For 4 patients, IgM was not detectable in the second serum sample, the earliest of which was drawn 50 days after onset of symptoms.

### Cumulative Diagnostic Parameters in Early Acute Illness

**Figure 2:**
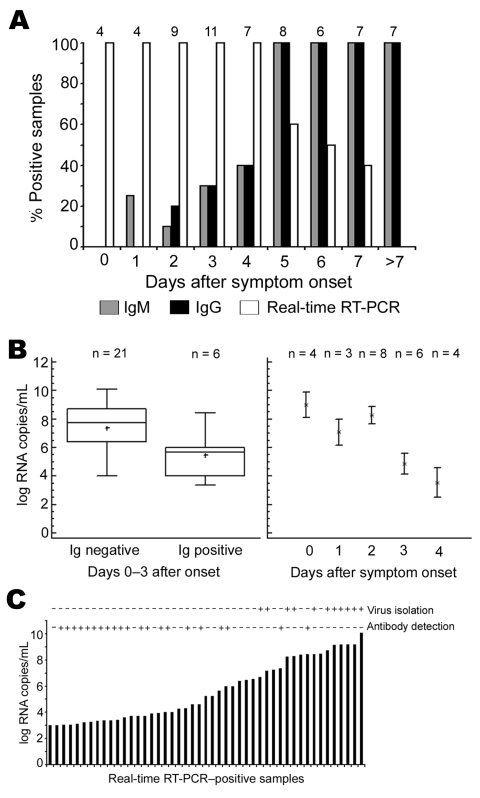
A) Rates of positive results from assays for immunoglobulin (Ig) M, IgG, and virus RNA, first 10 days of symptoms. Numbers above bars are numbers of samples (and patients). B) Left panel, viral loads in serum or plasma in antibody-negative, PCR-positive patients (n = 21, left column) and in antibody-positive, PCR-positive patients (n = 6, right column). All patients were sampled during first 3 days of symptoms. Right panel, viral loads in all antibody-negative, PCR-positive samples. Error bars represent interquartile ranges. C) Viral loads, antibodies, and virus isolation from 47 samples positive by reverse transcription–PCR (RT-PCR). Virus isolation + indicates isolation success as confirmed by cytopathogenic effect and direct immunofluorescence assay. Antibody detection + indicates an IgG or IgM titer >10 by immunofluorescence assay.

Of the 121 patients with complete histories, 45 (37.2%) had positive real-time RT-PCR results. To compare the efficiency of diagnostic methods in acute disease, we selected all samples collected during the first 10 days of symptoms from patients in whom CHIKV was confirmed by virus isolation or subsequent seroconversion (n = 63). RT-PCR was 100% positive up to day 4, regardless of antibody results (Figure 2). IgG and IgM were 100% positive from day 5 onward. IgM was detected only 1 day earlier than IgG. Overlapping RT-PCR and antibody results occurred from day1 to day 7. No positive RT-PCR result was found after day 7.

To assess whether CHIKV RNA detection may be influenced by antiviral antibodies, we compared RNA concentrations in seronegative (n = 21) and seropositive (n = 6) samples, all collected within the first 4 days of illness (Figure 2). Median RNA concentration was 9.85 × 10^7^ copies/mL in seronegative samples, which was significantly higher than the 2.35 × 10^5^ copies/mL found as median concentrations in seropositive samples (p<0.017, analysis of variance F-test). To characterize the course of viremia in absence of antibodies, we plotted viral loads of all antibody-negative, PCR-positive samples against sampling days. The highest concentrations were found in samples drawn on day 0 with a mean of 1.2 × 10^9^ RNA copies/mL (95% CI 8.2 × 10^8^–1.6 × 10^9^ copies/mL) (Figure 2). Viral loads in later samples continuously decreased.

### Efficiency of Virus Isolation

To reassess the diagnostic value of classic virus isolation techniques, we cultured 47 PCR-positive serum samples on Vero cells. All material had been frozen and thawed only 1 time before cell culture. Only 11 (23.4%) of 47 samples yielded a virus isolate. Virus was isolated only in samples containing >1 × 10^7^ RNA copies/mL (Figure 2). All culture-positive samples had been taken on or before the second day of fever, after a mean of 0.75 days from onset. Antibodies were not detectable in any of these samples, which suggests that infectivity in culture may be neutralized by antibodies.

### Dengue Virus Co-infections

Dengue virus is endemic to parts of the Indian Ocean region and resembles CHIKV infection. All serum samples received in the first half of 2006, including those from patients for whom travel and disease histories were incomplete, were tested for dengue virus. Only 3 of 368 patients had dengue IgG. Dengue IgM antibodies without IgG were found for 2 additional patients, which suggests acute infection. Both patients had traveled to Mauritius. One patient had dengue IgM and CHIKV RNA. This patient had a long period of fever (38°C–40°C for 11 days) and severe arthralgia. Another patient had dengue IgM and anti-CHIKV virus IgM and IgG. This patient had severe arthralgia for 1 month.

### E1 Protein Genotypes

**Figure 3:**
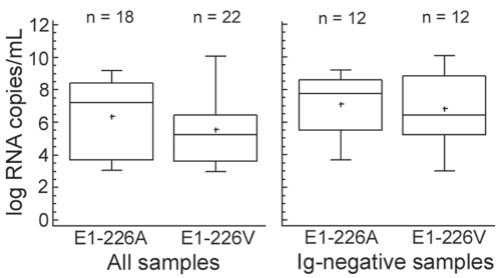
Viral loads for all PCR-positive samples (left panel) and immunoglobulin (Ig)–negative/PCR-positive samples (right panel), depending on types of mutation (alanine or valine at amino acid position 226 of the envelope 1 protein, as shown on the x-axis). Boxes represent the innermost 2 quartiles of data; horizontal line shows the mean; whiskers represent the outermost 2 quartiles.

We sequenced 40 PCR-positive samples, of which 18 (45%) showed the original E1-226A genotype and 22 (55%) had the E1-226V mutation (2 from Réunion Island, 20 from Mauritius, all sampled in the first half of 2006). Samples from India (n = 2) and from Sri Lanka (n = 2), which were collected in the second half of 2006, displayed the original E1-226A genotype. CHIKV viral loads among genotypes were compared (Figure 3). Median concentrations (1.6 × 10^7^ copies/mL for E1-226A; 1.6 × 10^5^ copies/mL for E1-226V) were not significantly different at the 95% confidence level. To exclude an influence of CHIKV antibodies on this result, we analyzed samples without antibodies separately. Again, the difference was not significant (226A samples (n = 12), 5 × 10^7^ copies/mL; 226V samples (n = 12), 2.5 × 10^6^ RNA copies/mL).

## Discussion

Chikungunya fever has become a major differential diagnosis, along with malaria and dengue fever, for ill travelers returning to Europe from the Indian Ocean region. Because chikungunya fever has similar symptoms but requires different management than malaria and dengue, laboratory testing for CHIKV is now part of routine clinical decision making. Our analysis of baseline virologic findings by using a modern repertoire of diagnostic tests comprised the largest cohort of travelers returning from sites of the current epidemic. Tourists are of particular interest because they are not seen in the context of community outbreaks, and thus, their diagnoses need individual laboratory confirmation. The large number of cases in our study enabled us to establish a gapless time course of virologic parameters by cumulating single- and dual-point laboratory determinations from individual infected travelers.

Cross-sectional laboratory data provided a good indication of the extent of this large outbreak of a usually underdiagnosed arbovirus infection in travelers. In the first half of 2006, as much as 24.4% clinically suspected cases of CHIKV infection could be confirmed. This predictive value of clinical diagnosis is impressive compared with that for suspected dengue virus infection in travelers (*29*,*30*). The decrease to 9.9% in the second half of 2006 is probably a reflection of the decreasing CHIKV activity in tourist destinations, along with a shift to the Indian subcontinent where tourism is less concentrated.

In the cumulative time course, anti-CHIKV IgG and IgM antibodies were detected soon after onset of symptoms. They were present in all patients in whom acute infection was confirmed after 5 days. IgG was present so early that additional testing for IgM provided only limited additional sensitivity for detecting acute cases. Most likely this finding was caused not by low sensitivity of our IgM assay but by an unusually early IgG response. One reason for early IgG findings could be lack of specificity of the applied immunofluorescence assay. However, we have shown recently that indirect immunofluorescence is a sensitive and specific method for determining antibodies against CHIKV (N. Litzba et al., unpub. data). Moreover, we have confirmed specificity of our immunofluorescence assay results by retesting a subset of serum samples by plaque-reduction neutralization test (data not shown); all antibody-positive samples subjected to this test had positive results. Finally, because most patients resided in Germany, where alphaviruses are not endemic, preexisting antibodies are unlikely. We must therefore assume that the IgG results are technically valid. An explanation for early IgG production would be the presence of unusually high presymptomatic viremia that provided an early antigen stimulus for antibody formation.

Cell culture in our study was not particularly diagnostic. It was rather insensitive (23.4%) compared with RT-PCR, and the presence of antibody seemed to prevent isolation of virus. In successful cultures, cytopathic effects were not observed before 3–4 days of incubation, often requiring at least 1 passage, which made time for culture longer than time to seroconversion. Nevertheless, culture is essential for more specific investigation such as virus neutralization tests, as well as for ecologic and basic research. For clinical virus detection, RT-PCR seemed to be the method of choice. Our study provides 2 alternative real-time RT-PCR protocols that have analytical performance comparable to that of major diagnostic PCR systems (*25*,*26*); each protocol has been clinically evaluated on our whole cohort.

Apart from practical diagnostic implications, our findings identify important questions for future research. As an example, courses of antibodies and viremia were different from those of dengue virus infections (*27*,*31*). Probably the most important difference was an early IgG response to CHIKV, compared with a clear delay of IgG (appearing after IgM) in most acute cases of primary dengue virus infection. For a substantial fraction of patients, dengue IgG is not detected until after 2 weeks of illness (*27*,*31*). One could speculate that the reason for this difference is the different tropism of each virus. Dengue virus infects predominantly monocytes (*31*) and thereby may induce a relevant, short-term immunosuppression (*32*). CHIKV probably does not share this tropism (*33*), although the primary target site of CHIKV infection is unclear. Whether co-infection with dengue and CHIKV would lead to altered symptoms is another question; 2 patients in our study had this co-infection. Their disease courses may be more severe or prolonged than those of most patients (*17*), but detailed and controlled studies on more patients are needed.

Data on viral load determined in this study are crucial for infection control. During their first few days of illness, our patients had tremendous virus concentrations, sometimes >10^9^ copies/mL of plasma. Such high viral loads are uncommon with other arboviral diseases (*34*–*36*) and would make CHIKV prone to nosocomial transmission, e.g., by needle-stick injury or mucous membrane exposure. Indeed such an event has already been documented (*23*). Blood and other body fluids of CHIKV patients should be considered infectious during the first week of symptoms. We cannot determine from this study whether the potential infectivity of CHIKV may also become important in the context of transfusion, because we analyzed only symptomatic patients. However, virus concentrations in our patients were already decreasing when symptoms were detected, and therefore a high viremia in presymptomatic patients seems likely. None of our patients had antibodies on day 0, and presymptomatic patients would therefore be highly infectious. The issue of transfusion transmissibility of CHIKV deserves intensive investigation. Targeted interventions like on Réunion Island, where blood donation was suspended during the epidemic and blood was imported from France (*37*), are not an option in India or Southeast Asia.

Why such a large epidemic of CHIKV infections could occur and why courses of disease were so unusually severe are unclear. A previous study recognized an A226V exchange in the E1 envelope glycoprotein for the first time in CHIKV and found it to increase over time in isolates from Réunion Island (*22*). In the related Semliki Forest virus, a corresponding mutation conferred a replication advantage in cholesterol-depleted insect cells (*24*), which for the virus would implicate an advantage in its reservoir. In addition to the above findings of Schuffenecker et al, we could confirm presence of the mutation on Mauritius, where a larger part of our patients had visited. However, viruses isolated in the late phase of our study (September to December 2006) from patients returning from India and Sri Lanka, respectively, did not show the mutation but were otherwise clearly related to the Indian Ocean strain. The significance of the mutation thus remains uncertain, and functional studies are needed. Confirmation of this or other genetic connections would provide another example of minor genetic differences that account for major changes of an arthropod virus. After the past and ongoing experiences with agents such as the coronavirus of severe acute respiratory syndrome and avian influenza viruses, the CHIKV epidemic provides more proof of the importance of research into the ecology of emerging and reemerging diseases.
